# Hybrid surgery can improve neurocognitive function in patients with internal carotid artery occlusion

**DOI:** 10.1038/s41598-023-50270-6

**Published:** 2023-12-21

**Authors:** Yudi Tang, Yunna Yang, Junqiang Feng, Yibo Geng, Yang Wang, Rong Wang, Dong Zhang, Jizong Zhao

**Affiliations:** 1 Department of Interventional Neuroradiology, Beijing Neurosurgical Institute, Capital Medical University, Beijing, China; 2 Department of Neurosurgery, Beijing Chaoyang Hospital, Capital Medical University, 8 Gongren Tiyuchang Nanlu, Chaoyang District, Beijing, 100020 China; 3 Department of Neurosurgery, Beijing Tiantan Hospital, Capital Medical University, Beijing, China; 4 Department of Neurosurgery, Beijing Hospital, Beijing, China

**Keywords:** Carotid artery disease, Cognitive neuroscience

## Abstract

Internal carotid artery occlusion (ICAO) is a relatively uncommon but important cause of transient ischaemic attack and cerebral infarction. Hybrid surgery (HS) improves cerebral perfusion, but its impact on neurocognitive function has been controversial. Patients with symptomatic chronic ICAO treated by hybrid surgery or medical treatment from 2016 to 2019 were included. We recorded and analysed the clinical characteristics, angiographic data, outcomes and cognitive status. Functional assessments, including the National Institutes of Health Stroke Scale (NIHSS), the Barthel Index, and a battery of neuropsychological tests, including the Mini-Mental State Examination (MMSE), Alzheimer Disease Assessment Scale–Cognitive Subtest (ADAS-cog), verbal fluency, and Colour Trail test Parts 1 and 2, were administered. Significant improvements in the ADAS (before, 7.5 ± 6.2 versus after, 5.2 ± 5.7; *P* = 0.022), MMSE (before, 25.5 ± 2.8 versus after, 28.1 ± 2.3; *P* = 0.013), and Colour Trail test Part 1 (before, 118.3 ± 26.5 versus after, 96.2 ± 23.1; *P* = 0.016) were observed six months after HS. Moreover, the abovementioned postprocedure scales were ameliorated in the HS group. This study found that in patients with multiple symptomatic ICAO and objective ipsilateral ischaemia, successful HS leads to improvement in the scores of three cognitive tests.

## Introduction

Patients with internal carotid artery occlusion (ICAO) have an increased risk of neurocognitive impairment, which may be caused by white matter lesions or ipsilateral ischaemia, especially in symptomatic ICAO^1^. The most common site for occlusion is the carotid artery bifurcation and the origin of the ICA. Carotid angioplasty and stenting (CAS) or carotid endarterectomy (CEA) are commonly used for treatment. Recently, hybrid surgery (HS), a novel approach that combines CAS and CEA, has been shown to be a feasible and effective procedure with a higher recanalization rate^2,3^. Additionally, previous studies have revealed that HS is a safe and effective method for ICAO patients^4^. Both CAS and CEA are effective in improving cognitive function in patients with carotid stenosis^5^. However, whether HS alleviates cognitive dysfunction remains a mystery.

Our study aimed to investigate the cognitive effects following the HS procedure. Therefore, we compared the baseline cognitive function of patients undergoing HS with those receiving conservative treatment (CTR). A diversity of cognitive scales was applied to measure different dimensions of cognitive status. Our study found, for the first time to our knowledge, that HS significantly improved cognitive function in symptomatic ICAO patients.

## Material and methods

### Patient selection

Patients with symptomatic chronic ICA occlusion admitted to the neurovascular ward in Beijing Tiantan Hospital from March 2016 to March 2019 were included in our study. Diagnosis of ICA occlusion was based on CTA, MRA or angiography with sufficient resolution. The study protocol was reviewed and approved by the local Ethics Committee (Beijing Tiantan Hospital, Capital Medical University). All methods in this study were carried out in accordance with the relevant guidelines and regulations, and all data in this study were obtained with informed consent from all subjects and/or their legal guardian(s).

First, patients suitable for hybrid surgery were selected^4^. The specific inclusion criteria were as follows: (1) the patients had recurrent ipsilateral ischaemic symptoms after medical treatment but had no new ischaemic stroke (≥ 4 weeks); (2) the ipsilateral middle cerebral artery was patent, and no smoke-like blood vessel was present; cerebral haemodynamics were impaired with ‘misery perfusion’ or in stage II haemodynamic failure, including an increase in the mean transit time (MTT) more than 4 s, a decreased cerebral blood flow (CBF) ratio (symptomatic side/asymptomatic side < 0.95) and increasing oxygen extraction fraction (OEF) ratio (ipsilateral side/contralateral side > 1.13) on preoperative perfusion CT (CTP)/MR imaging (MRI) or positron emission tomography (PET); and the proximal occlusion of the ICA had no stump or tamper. The exclusion criteria were as follows: the presence of severe systemic disease that prevented surgery and anaesthesia; the presence of heart disease (unstable angina or acute myocardial infarction), bleeding disorder, or a contraindication to aspirin, clopidogrel, heparin, or iodine contrast; and older patients with end-stage disease or in poor neurologic condition. In addition, patients who achieved CAS and those who had CEA or other surgeries were not selected.

Second, because of the influence on cognitive assessment, patients had any of following points: (1) the blockage was formed by a tangle of tiny vessels, (2) contralesional MCA stenosis > 50%, (3) vascular surgery performed on the responsible vessel, (4) intracranial surgery, (5) severe systemic or neuropsychiatric disease, (6) massive cerebral infarction and NIHSS ≥ 8, (7) educational level below elementary school, (8) pretreatment modified Rankin score > 3, (9) ischaemic stroke within 4 weeks, and (10) lack of complete clinical records.

### Grouping of patients

The patients who received successful HS were divided into the HS group, while those receiving CTR were placed in the CTR group. Each group was further divided into baseline and posttreatment states to compare the effects of the respective treatments.

### Neurocognitive function evaluation

Cognitive function evaluations were conducted within 7 days before enrolment and 6 months after enrolment by an independent clinical psychologist and a physician who were unaware of the intervention outcomes. The initial assessments focused on the severity of neurological and cognitive impairment. The evaluation included the Mini-Mental State Examination (MMSE), Alzheimer Disease Assessment Scale (ADAS), National Institutes of Health Stroke Scale (NIHSS), Barthel Index, Verbal Fluency Test (VFT), and Colour Trails Test (CTT). The difference between the pre- and posttreatment values (value change) and the percentage of the difference from the pretreatment values (percentage change) were used to indicate the degree of neurocognitive change.

### CEA-CEA hybrid procedure

The specifics of the hybrid surgery procedure were described previously^4^. In summary, all patients in the HS group were administered aspirin and clopidogrel for at least 5 days before the surgical procedure. Initially, the patients underwent standard CEA under general anaesthesia. Subsequently, if minimal or no blood backflow was observed, CEA was performed in a hybrid operating room. Ultimately, a residual diameter stenosis of less than 20% was considered successful HS.

### Post-HS management

A postoperative CT scan was conducted to detect any intracranial haemorrhage or new ischaemic lesions, and blood pressure was typically maintained at less than 140/90 mmHg for 3 days. For patients without hypertension, strict control of blood pressure was maintained at less than 120 mmHg. Patients who received stent placement were prescribed aspirin (100 mg/day), clopidogrel (75 mg/day), and atorvastatin calcium (20 mg/day) for a minimum of 3 months, followed by lifelong administration of a single antiplatelet agent and atorvastatin calcium.

### CTR management

Patients undergoing CTR were strictly instructed to stop smoking and drinking. They were prescribed aspirin (100 mg/day), clopidogrel (75 mg/day), and atorvastatin calcium (20 mg/day) tablets. Furthermore, the patients' blood pressure, blood sugar, and blood lipids were continually monitored and managed.

### Cerebral perfusion evaluation

Cerebral perfusion was assessed using CTP (GE-Health care Discovery CT 750HD) and analysed automatically with RAPID software (iSchemaView, Menlo Park, CA). A comparison of the MTT and time to peak (TTP) before and after treatment was used to evaluate changes in cerebral perfusion. The value change and percentage change were used to indicate the degree of cerebral perfusion change.

### Follow-up

During the first three months after surgery, CTA, CTP, or MRP were performed to assess the degree of revascularization. Subsequently, DSA was conducted, followed by survival, neurological, and cognitive evaluations six months after surgery.

### Statistics

All statistical analyses were conducted using SPSS V25.0. Continuous or discrete data are presented as the mean ± standard deviation or as counts and percentages, respectively. The chi-square test or Fisher's exact test was performed for categorical variables as appropriate. Normally distributed continuous variables were compared using Student’s t test, while nonnormally distributed variables were compared using the Mann–Whitney U test. Correlations were examined using Pearson’s or Spearman’s method according to the distribution type. A *p* value < 0.05 was considered statistically significant.

## Results

Overall, 813 consecutive patients with ICAO admitted to the neurovascular ward in Beijing Tiantan Hospital were screened, and 106 patients were selected for our study. However, 26 of them were excluded (11 without complete clinical records because of loss to follow-up, 10 with failed vascular surgery on the responsible vessel in the past, 2 with contralesional MCA stenosis > 50%, 1 with massive cerebral infarction in the past, and 1 without complete clinical records because of a lack of preoperative MRI and CT scans). Briefly, 80 patients were included in this study: 40 patients in the hybrid surgery (HS) group and 40 patients in the conservative treatment (CTR) group.

### Patient characteristics

The baseline characteristics of the two groups, including sex, age, smoking, excessive drinking, hypertension, diabetes, hyperlipidaemia, coronary heart disease, peripheral occlusive vascular disease, left intracranial artery occlusion, opposite intracranial artery stenosis, cerebral infarction and chronic renal insufficiency, are shown in Table 1. The differences in all variables between the two groups were not significant.

**Table 1:** Baseline and hospital characteristics of patients undergoing HS and CTR.

Variables	HS (n = 40)	CTR (n = 40)	t/X2	p
Male, n (%)	32 (80.0)	30 (75.0)	0.633	0.273
Age (years), mean ± SD	65.1 ± 8.4	63.1 ± 8.2	0.524	0.249
Smoking, n (%)	6 (15.0)	8 (20.0)	0.132	0.347
Drinking, n (%)	6 (15.0)	7 (17.5)	0.143	0.372
Hypertension, n (%)	18 (45.0)	15 (37.5)	0.160	0.169
Diabetes, n (%)	16 (40.0)	19 (47.5)	0.341	0.197
Hyperlipidaemia, n(%)	12 (30.0)	9 (22.5)	0.156	0.139
Coronary heart disease, n (%)	10 (25.0)	8 (20.0)	0.162	0.178
POVD	5 (12.5)	7 (17.5)	0.142	0.195
Left ICAO, n (%)	11 (27.5)	13 (32.5)	0.196	0.653
opposite ICAS > 50%, n (%)	5 (12.5)	6 (15.0)	0.326	0.498
Cerebral infarction, n (%)	31 (77.5)	32 (80.0)	0.361	0.472
CRI, n (%)	0 (0)	3 (7.5)	0.213	0.315

*HS* hybrid surgery; *CTR* conservative treatment; *SD* standard deviation; *n* number; *POVD* peripheral occlusive vascular disease; *ICAO* intracranial artery occlusion; *ICAS* intracranial artery stenosis; *CRI* chronic renal insufficiency.

### Cognitive improvement

Among the HS group, significant improvements in the MMSE (pre 25.5 ± 2.8 vs. post 28.1 ± 2.3, *p* = 0.013), colour trials test A (pre 118.3 ± 26.5 vs. post 96.2 ± 23.1, *p* = 0.016) and ADAS (pre 7.5 ± 6.2 vs. post 5.2 ± 5.7, *p* = 0.022) were achieved after HS (Table 2). However, no significant change in any test neurocognitive parameter was confirmed in the CTR group. The NIHSS, verbal fluency and Barthel index were stationary among the two groups at six months postprocedure (*p* > 0.05).

**Table 2:** Differences in neurocognitive and neurologic function from baseline to 6 months postprocedure among groups.

Variable	HS			CTR		
	Baseline	Post-HS	*P*	Baseline	Posttreatment	*p*
MMSE score, mean ± SD	25.5 ± 2.8	28.1 ± 2.3	**0.013**	24.6 ± 5.4	25.2 ± 4.8	0.425
NIHSS score, mean ± SD	0.7 ± 0.5	0.5 ± 0.6	0.159	0.6 ± 0.4	0.6 ± 0.3	NA
Barthel index, mean ± SD	96.4 ± 7.9	97.3 ± 4.8	0.309	95.9 ± 6.2	97.2 ± 4.1	0.303
Colour trails test A, mean ± SD	118.3 ± 26.5	96.2 ± 23.1	**0.016**	131.3 ± 31.1	127.3 ± 27.7	0.776
Colour trails test B, mean ± SD	189.5 ± 49.3	167.1 ± 47.8	0.163	179.7 ± 52.1	185.1 ± 54.6	0.387
Verbal fluency test, mean ± SD	26.7 ± 11.2	27.5 ± 10.4	0.935	27.2 ± 9.3	26.3 ± 7.5	0.995
ADAS score, mean ± SD	7.5 ± 6. 2	5.2 ± 5.7	**0.022**	7.9 ± 6.7	8.3 ± 7.2	0.263

Significant values are in bold.

*MMSE* Mini-Mental State Examination; *NIHSS* National Institutes of Health Stroke Scale; *ADAS* Alzheimer’s Disease Assessment Scale; *HS* hybrid surgery; *CTR* conservative treatment; *SD* standard deviation.

After 6 months, significant improvements in the MMSE (HS 28.1 ± 2.3 vs. CTR 25.2 ± 4.8, *p* = 0.015), colour trials test A (HS 96.2 ± 23.1 vs. CTR 127.3 ± 27.7, *p* = 0.013) and ADAS (HS 5.2 ± 5.9 vs. CTR 8.3 ± 7.2, *p* = 0.014) were achieved in the HS group on the basis of similar cognitive assessment before treatment (Table 3). The differences in NIHSS, verbal fluency and the Barthel index between the HS and CTR groups were not significant (*P* > 0.05).

**Table 3:** Differences in neurocognitive and neurologic function at 6 months postprocedure between the two groups.

Variable	Preprocedure			Postprocedure		
	HS	CTR	*P*	HS	CTR	*p*
MMSE score, mean ± SD	25.5 ± 2.8	24.6 ± 5.4	0.951	28.1 ± 2.3	25.2 ± 4.8	**0.015**
NIHSS score, mean ± SD	0.7 ± 0.5	0.6 ± 0.4	0.836	0.5 ± 0.6	0.6 ± 0.3	0.237
Barthel index, mean ± SD	96.4 ± 7.9	95.9 ± 6.2	0.913	97.3 ± 4.8	97.2 ± 4.1	0.527
Colour trails test A, mean ± SD	118.3 ± 26.5	131.3 ± 31.1	0.276	96.2 ± 23.1	127.3 ± 27.7	**0.013**
Colour trails test B, mean ± SD	189.5 ± 49.3	179.7 ± 52.1	0.741	167.1 ± 47.8	185.1 ± 54.6	0.205
Verbal fluency test, mean ± SD	26.7 ± 11.2	27.2 ± 9.3	0.995	27.5 ± 10.4	26.3 ± 7.5	0.872
ADAS score, mean ± SD	7.5 ± 6. 2	7.9 ± 6.7	0.971	5.2 ± 5.7	8.3 ± 7.2	**0.014**

Significant values are in bold.

*MMSE* Mini-Mental State Examination; *NIHSS* National Institutes of Health Stroke Scale; *ADAS* Alzheimer’s Disease Assessment Scale; *HS* hybrid surgery; *CTR* conservative treatment; *SD* standard deviation.

### Cerebral perfusion improvement

In the HS group, the mean MTT was 10.74(SD: 1.42)s before treatment and 8.16(SD: 0.67)s after treatment, and the TTP was 18.64(SD: 0.86)s before treatment and 13.66(SD: 1.31)s after treatment. In CTR group, the mean MTT was 11.87(SD: 1.39)s before treatment and 11.84(SD: 1.34)s after treatment, and the TTP was 18.38(SD: 0.59)s before treatment and 18.37(SD: 0.60)s after treatment.

**Figure 1:**
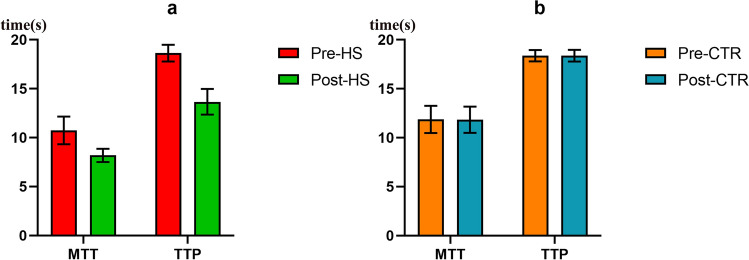
Mean MTT and TTP before and after treatment in the two groups (1a. HS group; 1b. CTR group).

Both the MTT and TTP were significantly improved (MTT: *p* < 0.001; TTP: *p* < 0.001) in the HS group and were not significantly changed (MTT: *p* = 0.314; TTP: *p* = 0.892) in the CTR group after treatment (Fig. 1).

### Correlation between cognitive and cerebral perfusion in the HS group

Before HS, both the MTT and TTP were negatively correlated with the VFT (MTT: r = − 0.373, *p* = 0.042; TTP: r = − 0.367, *p* = 0.046) and positively correlated with the NIHSS (MTT: r = 0.458, *p* = 0.011; TTP: r = 0.460, *p* = 0.011), and the TTP was negatively correlated with the Barthel index (r = − 0.382, *p* = 0.037). After HS, both the MTT and TTP were negatively correlated with the Barthel index (MTT: r = − 0.427, *p* = 0.019; TTP: r = − 0.402, *p* = 0.028). More details are shown in Table 4.

**Table 4:** Correlation between neurocognitive evaluation and cerebral perfusion in pre-HS, post-HS, value change and percentage change.

		MMSE	Barthel	NIHSS	CTT A	CTT B	VFT	ADAs
MTT	Pre	− 0.013	− 0.326	0.458	− 0.346	0.205	− 0.373	− 0.230
	*p*	0.945	0.079	0.011	0.061	0.278	0.042	0.221
	Post	− 0.312	− 0.427	0.304	− 0.079	− 0.262	− 0.223	0.228
	*p*	0.093	0.019	0.102	0.677	0.161	0.235	0.226
	Change^#^	0.063	− 0.233	0.342	− 0.243	0.388	− 0.226	− 0.110
	*p*	0.741	0.215	0.065	0.195	0.034	0.230	0.563
	Change*	0.149	− 0.168	−	− 0.155	0.305	− 0.225	0.046
	*p*	0.432	0.375	−	0.413	0.101	0.233	0.809
TTP	Pre	− 0.302	− 0.382	0.460	− 0.284	− 0.163	− 0.367	− 0.056
	*p*	0.105	0.037	0.011	0.128	0.390	0.046	0.770
	Post	− 0.322	− 0.402	0.329	0.030	− 0.335	− 0.172	0.242
	*p*	0.083	0.028	0.075	0.873	0.070	0.362	0.197
	Change^#^	− 0.004	− 0.275	0.040	0.370	− 0.371	− 0.100	− 0.022
	*p*	0.985	0.141	0.834	0.044	0.044	0.600	0.907
	Change*	0.067	− 0.211	–	0.403	− 0.435	− 0.124	0.181
	*p*	0.727	0.263	–	0.027	0.016	0.515	0.338

Change^#^: value change [pre-HS value – post-HS value].

Change*: percentage change [(pre-HS value – post-HS value)/pre-HS value].

*MMSE* Mini-Mental State Examination; *NIHSS* National Institutes of Health Stroke Scale; *ADAS* Alzheimer’s Disease Assessment Scale; *HS* hybrid surgery; *MTT* mean transit time; *TTP* time to peak.

The MTT was positively correlated with the CTT B (r = 0.388, *p* = 0.034) in the value change, but there was no significant (*p* = 0.101) correlation in the percentage change. The TTP was positively correlated with the CTT A and negatively correlated with the CTT B in both the value change (CCT A: r = 0.370, *p* = 0.044; CCT B: r = − 0.371, *p* = 0.044) and percentage change (CCT A: r = 0.403, *p* = 0.027; CCT B: r = − 0.435, *p* = 0.016). More details are shown in Table 4.

### Correlation of cognitive function before and after HS

A strong positive correlation is shown in all tests between pre- and post-HS (CTT A: r = 0.706, *p* < 0.001; CTT B: r = 0.377, *p* = 0.010; MMSE: r = 0.824, *p* < 0.001; Barthel: r = 0.799, *p* < 0.001; NIHSS: r = 0.610, *p* < 0.001; VFT: r = 0. 925, *p* < 0.001; VFT: r = 0. 671, *p* < 0.001).

### Symptomatic complication

In the HS group, 3 patients had symptomatic complications (1 with hoarseness, 1 with hyperperfusion syndrome with minor subarachnoid haemorrhage, and 1 with ICA reocclusion). In the CTR group, 1 patient experienced transient ischaemic attack (TIA), and 1 patient experienced a minor ischaemic stroke.

## Discussion

Neurocognitive outcomes following ICAO have been significantly affected by the lack of effective treatment^6^. Available options include carotid endarterectomy, carotid artery stenting, and hybrid surgery, which combines vascular surgery with a catheter-based interventional approach^7^. Fisher noted that carotid artery disease may lead to cognitive impairment or a dementia state based on necropsy cases and suggested that restoring blood supply could reverse the condition ^8^. The mechanisms of cognitive impairment in patients with ICAO include embolization, hypoperfusion, and disseminated demyelinating lesions in the brain's white matter^9,10^. Both CEA and CAS have been shown to partially reverse the pathological process and improve cognitive impairment. Recently, hybrid surgery has been performed for minimally invasive procedures and has achieved a higher angiographic success rate and fewer complications than CEA or CAS alone^11^. Therefore, HS should demonstrate similar results in improving cognitive impairment. However, previous studies have mainly focused on the correlation between cognition recovery and CEA and CAS, and there is a lack of research on the correlation with HS^12^. Our results indicate that neurocognitive function was improved after successful HS, which is consistent with Fisher's original hypothesis. However, whether HS can lead to better cognitive function improvement than CAS and CEA needs to be further explored.

Sridharan et al.^13^ analysed previous studies and found that cognitive improvement was uncertain after CEA among patients with asymptomatic carotid stenosis. Similarly, the cognitive improvement in asymptomatic patients who received CAS was inconsistent^14,15^. Consequently, our project focused on symptomatic patients with stage II haemodynamic failure. Fan et al.^16^ observed that all patients achieved intraoperative recanalization (thrombolysis in cerebral infarction [TICI] stage IIb or III) and found that 87.5% (35/40) of patients experienced relief from symptoms after HS. Based on this, the amelioration of postoperative perfusion was confirmed in our previously published research^4^.

In the present study, we demonstrated the beneficial effect of revascularization on cognition. We compared neurocognitive function between HS and CTR among ICAO patients and revealed that HS remarkably improved cognitive performance in terms of mental status, dementia and cognitive flexibility with the help of several scales. Both the MMSE and ADAS are two of the most common questionnaires for cognitive impairment^17^. In addition, the colour trial test was used to measure attention, visual scanning and working memory^18^. In addition, the majority of the population with carotid stenosis consists of patients with minimal cognitive impairment^19^. Therefore, examinations such as the MMSE, ADAS, and CTT A, which are domains containing psychomotor speed/reaction time, attention, memory, and visuoconstructional organization, can be more sensitive in showing cognitive improvement after HS^19^. Although the NIHSS, Barthel, CTT B, and VFT did not show significant improvement, they all showed a certain degree of correlation with cerebral perfusion. Thus, we speculate that improvements in other cognitive functions can be demonstrated through more detailed testing. We believe that HS improves cognitive function in ICAO patients by increasing cerebral perfusion.

Although the MTT was positively correlated with the CCT B in value change, the TTP was negatively correlated with the CCT B in both value change and percentage change. For this confusing result, we have the following hypothesis: 1. Inappropriate test interpretation: The same numerical increase does not represent a consistent increase in cognitive function. The higher the preoperative value, the more likely it is to change the value. 2. Injury of chronic cerebral hypoperfusion (CCH): The greater the increase in the TTP, the more severe the underlying CCH. CCH causes brain injury through immune inflammatory injury^20^, oxidative stress injury^21^, vascular endothelial dysfunction^22^ and neurotransmitter changes^23^. These injuries may take more time to recover or may be irreversible. 3. Asymptomatic cerebral hyperperfusion syndrome (CHS): Larger TTP changes mean greater increases in cerebral perfusion over a short period of time. Although there is a lack of research in asymptomatic CHS, it is not surprising that it can occur only in a minor part of the brain according to the pathogenesis mechanism of CHS. A similar case has been reported^24^. In addition, there was a strong positive correlation between the CTT B before and after HS, indicating that patients with a better CTT B score before HS usually had a better score after HS.

In terms of cognitive functioning, the results could be influenced by various factors, such as the patients' characteristics, initial intelligence or memory, self-healing, and the so-called ‘practice effect’. The comparison of demographics and characteristics between the HS and CTR groups showed similar baseline characteristics before treatment (Table 1). Furthermore, the comparison of the CTR group between pre- and postprocedure balanced the ‘practice effect’ (Table 2).

### Limitations

This study only focused on ICAO patients who met the HS criteria without other diseases that can affect cognitive function. Although patients receiving CTR in this study did not show cognitive improvement, this does not mean that all ICAO patients, especially complicated cases, will not improve after CTR. In addition, there were also several limitations to this study. First, it was challenging to precisely define the duration of occlusion, which could have impacted the reversibility of cognition after treatment. Second, it was a retrospective study conducted at a single centre, with a limited sample size. Third, the strict inclusion and exclusion criteria may limit the applicability of the findings to all patients. In addition, the assessment of long-term neurocognitive recovery was hindered by the 6-month follow-up period. The results of this study need to be further verified in a large prospective longitudinal cohort.

## Conclusion

This study found that in patients with multiple symptomatic ICAO and objective ipsilateral ischaemia, successful HS leads to improvement in the scores of three cognitive tests.

## Data Availability

The data that support the findings of this study are available upon request from the corresponding author, YY.
